# Therapeutic Effects of Acetone Extract of *Saraca asoca* Seeds on Rats with Adjuvant-Induced Arthritis via Attenuating Inflammatory Responses

**DOI:** 10.1155/2014/959687

**Published:** 2014-03-04

**Authors:** Mradu Gupta, Saumyakanti Sasmal, Arup Mukherjee

**Affiliations:** ^1^Dravyaguna Department, Institute of Post Graduate Ayurvedic Education and Research, 294/3/1, A.P.C. Road, Kolkata, West Bengal 700009, India; ^2^Department of Chemical Technology, University College of Science and Technology, Calcutta University, Kolkata, West Bengal 700009, India

## Abstract

*Saraca asoca* has been traditionally used in Indian system for treatment of uterine, genital, and other reproductive disorders in women, fever, pain, and inflammation. The hypothesis of this study is that acetone extract of *Saraca asoca* seeds is an effective anti-inflammatory treatment for arthritis in animal experiments. The antiarthritic effect of its oral administration on Freund's adjuvant-induced arthritis has been studied in Wistar albino rats after acute and subacute toxicities. Phytochemical analysis revealed presence of high concentrations of phenolic compounds such as flavonoids and tannins, while no mortality or morbidity was observed up to 1000 mg/kg dose during acute and subacute toxicity assessments. Regular treatment up to 21 days of adjuvant-induced arthritic rats with *Saraca asoca *acetone extract (at 300 and 500 mg/kg doses) increases RBC and Hb, decreases WBC, ESR, and prostaglandin levels in blood, and restores body weight when compared with control (normal saline) and standard (Indomethacin) groups. Significant (*P* < 0.05) inhibitory effect was observed especially at higher dose on paw edema, ankle joint inflammation, and hydroxyproline and glucosamine concentrations in urine. Normal radiological images of joint and histopathological analysis of joint, liver, stomach, and kidney also confirmed its significant nontoxic, antiarthritic, and anti-inflammatory effect.

## 1. Introduction

Arthritis affects around 0.5–1% of the world population with more women being affected than men. The immune system is a well-organized and well-regulated structure. The deregulation of the immune system may lead to the development of autoimmune diseases such as Rheumatoid arthritis (RA) which is a prototype of the groups of illnesses with chronic systemic disorders with destructive inflammatory polyarticular joint potentially resulting in progressive destruction of articular and periarticular structure. Persistent inflammation produces swollen joints with severe synovitis, decreased nociceptive threshold, and massive subsynovial infiltration of mononuclear cells, which along with angiogenesis leads to pannus formation. Expansion of the pannus induces bone erosion and cartilage thinning, leading to the loss of joint function in due course. This results in a high degree of morbidity and disturbed daily life of the patient. Corticosteroids have not been able to fully control the incidence because of its limitations and risk of side effects. Many patients and practitioners are seeking alternative approach to provide an effective cure in the treatment of arthritis and to overcome the serious drawbacks such as gastrointestinal bleeding on treatment with corticosteroids. Hence, there is an urgent need to find safer drugs for the management of rheumatoid arthritis which is linked to inflammation of joints [1].

Many herbal formulations in the form of a single drug or compound drugs have been used for the treatment of joint pain, fever, and inflammation since ancient times as per the Indian system of Ayurvedic medicine. *Saraca asoca* has been traditionally used in the Indian system from time immemorial for the treatment of uterine, genital, and other reproductive disorders in women, ailments of urogenital tract, fever, pain, and so forth. Its properties have been mentioned in the ancient Ayurvedic text Charak Samhita under the Vedanasthapan (analgesic, antipyretic, and anti-inflammatory) category [2–5]. The legumes of *Saraca asoca* are 6–10 inches long containing 4–8 grey dicotyledonous seeds like a chest nut. The seeds are 3–5 cm long with average diameter of 8-9 cm, smooth surface, ellipsoid-oblong, and compressed. The seed coating is brown or slightly black in colour while sun-dried seeds are dark brown coloured having a smooth surface with hard texture. The stem bark part of this plant contains tannin, catechol, sterol, organic calcium compounds, essential oil, haematoxylin, a ketosterol, a crystalline glycosidal constituent, saponin, organic iron compound, leucocyanidin, and quercetin. The pharmacological activities of stem bark are uterogenic, antibacterial, oxytocic, antitumour, anticancer, and antiprogestational.

Saracin, a seed integument lectin from *Saraca indica,* is highly specific for binding N-acetylneuraminyl-N-acetyllactosamine [Neu5Ac-*α*-(2–6)/(2-3)-d-Gal-*β*-(1–4)-d-GlcNAc]. This lectin has been found to be mitogenic for human lymphocytes, and this mitogenic activity could be inhibited in presence of fetuin. Further, treatment with Saracin could induce secretion of IL-2 in a culture of resting human peripheral blood mononuclear cells (PBMC) after 48 h. Saracin has a higher affinity for the CD8+ than CD4+ T cells as revealed by FACS analysis [6]. *Saraca asoca* inhibited all TFs/DNA interactions even if at different concentrations. The medicinal plant extracts exhibiting inhibitory activity on cell proliferation should undergo analysis for possible antitumor activity, while extracts displaying inhibition of TFs/DNA interactions without effects on cell growth kinetics might be employed to control TFs-dependent gene expression without cytotoxic effects, including the case of inflammatory processes involved in relevant human pathologies, such as rheumatoid arthritis and cystic fibrosis [7].

The scientific pharmacological evaluation of the analgesic, antipyretic, and acute anti-inflammatory activities of the acetone extract of seeds of *Saraca asoca* has given significant and positive results during animal experimentation [8]. Therefore, its antiarthritic pharmacological action was evaluated on animals following the adjuvant test to find out its chronic anti-inflammatory effect which could validate the possible usage of these seeds as an effective nonsteroidal anti-inflammatory antiarthritic drug having the property of antioxidant, immune modulator, analgesic, and so forth.

## 2. Materials and Methods

The pharmacognostical, chemical, and experimental studies were carried out in the laboratory of the Department of Dravyaguna (Medicinal Plant Pharmacology) at the Institute of Post Graduate Ayurvedic Education & Research, Kolkata. The acute and subacute toxicity and adjuvant antiarthritis studies of acetone extract of the seeds of *Saraca asoca* were done on rodent animals after getting approval from the Institutional Animal Ethical Committee (IAEC) in the animal house of IPGAE&R Kolkata (registration number 1180/ac/08/CPSEA dated 27.03.2008 of CPCSEA) according to the guidelines of CPCSEA.

### 2.1. Plant Materials

The seeds of *Saraca asoca* were collected from the medicinal plant garden of Narendrapur Ramakrishna Mission, Kolkata, and the State Government Herbal Garden at Kalyani, West Bengal, India, in the month of July. The identification of seeds was done by the botanist at the Botanical Survey of India, Howrah, India, vide Ref. number BSI/CNH/AD/Tech./2010 and Sample Reg. number AS-01. An authentic herbarium specimen was deposited in the herbarium museum of the Department of Dravyaguna at IPGAE&R, Kolkata, for future reference.

### 2.2. Chemicals

The CFA reagent was purchased from M/s Sigma Aldrich, USA, while hydroxyproline and quercetin were purchased from M/s SRL, India, and indomethacin was purchased from M/s Jagsonpal Pharmaceuticals Ltd., India. All other chemicals used in the study were of analytical grade.

### 2.3. Pharmacognostical Study

The macroscopic and microscopic examination of the seeds were done for the purpose of standardization. Coarse powder (number 40 mesh) was used for extraction and fine powder was used to ascertain the macroscopic and microscopic pharmacognostical characteristics under high resolution microscope (Dewinter, Italy) at the Department of Dravyaguna of IPGAE&R according to established procedures [9, 10].

### 2.4. Method of Extraction

The seeds were washed and cleaned thoroughly to remove any extraneous matter and kept initially under sunlight covered with fine net for 5 hours. They were dried under shade for 10 days taking all precautions to prevent them from any contamination and other foreign matters. The completely dried whole seeds were powdered with a grinding machine (Hammer mill), passed through a number 40 mesh sieve, and stored in airtight containers for experimental purposes. The powder of research drug was subsequently extracted sequentially in petroleum ether (60°–80°), chloroform, acetone, methanol, and water in a Soxhlet's extractor and then filtered. The acetone extract was concentrated under vacuum in a rotary evaporator to yield semisolid mass. This was further dried under a vacuum oven drier to give solid residue and preserved in refrigerator below 10°C for experimental work.

### 2.5. Physiochemical and Phytochemical Analysis

The various important physiochemical parameters such as pH value, extractive values, moisture content, ash values, total flavonoid and phenol content, seed oil properties, and fluorescence of the powdered seeds were estimated using standard methods. The presence of important phytochemical constituents such as phenols, flavonoids, tannins, and saponins was assessed using standard techniques [11, 12].

### 2.6. Animals

Swiss albino mice of either sex, weighing about 20–30 gm, and albino (Wistar) rats of either sex, weighing about 120–130 gm, were used for different *in vivo* evaluation. All animals were procured from the Government of West Bengal approved breeder, M/s Satyacharan Ghosh, Kolkata, and housed under standard environmental conditions with fixed 12 h light/dark cycles and a temperature of approximately 25°C in animal house of IPGAE&R. The animals were kept in standard polypropylene cages and provided with food (standard pellet diet) and water *ad libitum*. These animals were acclimatized for a period of 14 days prior to performing any experiments. All experimental protocols were approved by the Institutional Animal Ethics Committee [13].

### 2.7. Acute Toxicity Study

Acute toxicity study was carried out on healthy Swiss albino mice following OECD guideline 423 [14]. The animals of both sexes were selected by random sampling technique and divided into 5 groups of 3 animals each. A single oral dose of the extract was administered orally at the level of 100 mg, 300 mg, 500 mg, 700 mg, and 1000 mg/kg body weight, respectively. All the animals were observed for appearance of toxic symptoms including muscle spasm, loss of righting reflex, tremors, behavioural changes, locomotion, convulsions, and mortality for 1, 2, 4, 8, and 24 h. Long-term supervision was continued for a period of 14 days for observing any occurrence of toxic symptoms and mortality [15].

### 2.8. Subacute Toxicity Study

The six Wistarrats were dosed daily, starting at 300 mg/kg body weight (the expected therapeutic level) and increased stepwise every two to three days on to 400, 500, 600, 700, 800, 900, and 1000 mg/kg dosage, respectively, until toxic signs were observed. Hematological and biochemical monitoring were carried out and blood level of the compound was checked to ensure its absorption. The animals were maintained at the maximum tolerated dose for a period of two to three weeks to allow development of any pathological changes, killed thereafter, and subjected to full pathological and histological examination. The purpose of this test was to determine the maximum tolerated dose and to ascertain the nature of toxic reactions, so that suitable chronic toxicity studies can be designed to fully evaluate the toxic potential of the compound. The clinical symptoms of each animal in terms of behavioral patterns detailed in the acute toxicity section mentioned above were also observed in all groups [15, 16].

### 2.9. Evaluation of Antiarthritic Activity

FCA-induced arthritis model in rats is suggested as the most suitable model of chronic and subchronic inflammation. Rat adjuvant arthritis is an experimental model of polyarthritis which has been widely used for preclinical testing of numerous antiarthritic agents which are either under preclinical or clinical investigation or are currently used as therapeutics in this disease [17–19].

Animals like Wistar rats were randomly divided into four groups of six animals each (*n* = 6). The control group received 10 mL/kg of vehicle in which the extract was going to be suspended while the standard group received indomethacin (2.5 mg/kg p.o) as the reference standard. The third and fourth groups of animals were administered acetone extract of *Saraca asoca *(AE) at a dose of 300 mg/kg and 500 mg/kg, respectively. Arthritis was induced by a single subplanter injection of 0.1 mL of complete Freund's adjuvant (CFA) containing 1.0 mg dry heat-killed *Mycobacterium tuberculoi *per mL sterile paraffin oil into foot pad of left hind paw of male Wistar rats. Drug treatment was started in all the groups from the day of adjuvant injection (0 day) 30 min before adjuvant injection and continued till 21st day. The swelling in hind paw (oedema) and paw ankle joint of left foot was periodically examined using screw gauge [20]. The arthritic effect and other clinical symptoms were observed in all the animals up to 28 days. It was observed during the experiment that rats of standard group expired within a week when administered indomethacin at the dose of 10 mL/kg daily due to gastric ulceration and other toxicities in the body. Therefore, the dose of indomethacin was lowered and after several rounds of trial, the dose of 2.5 mg/kg body weight was selected which provided highly significant results and no mortality of animals. The following parameters were evaluated in this study up to 21 days.

#### 2.9.1. Measurement of Body Weight and Oedema of Left Paw and Ankle Joint

The diameter of left paw and left ankle joint was measured in mm on 0, 2nd, 5th, 7th, 9th, 11th, 14th, 18th, and 21st days by using screw gauge and the body weight of animals was measured by digital balance. The mean changes in weight and diameter of paw oedema and ankle joint as well as their percentage inhibition with respect to control group were calculated on the 7th day, 14th day, and 21st day, respectively.

#### 2.9.2. Routine Haematology and Estimation of the Prostaglandin in Blood of the Rats

The rats were anaesthetized under light ether anaesthesia and blood was collected by retroorbital puncture for estimation of serum parameters such as Hb, RBC, WBC, ESR, and prostaglandin (E_2_ EIA Kit—Monoclonal Cayman Chemical Item number 514010) by using various diagnostic kits on the 14th day and 21st day which were compared with the data obtained from untreated rats before injection of adjuvant to the rats.

#### 2.9.3. Estimation of Hydroxyproline and Glucosamine in Urine Sample of Rats

This procedure is used to assess the urinary excretion of connective tissue metabolites such as hydroxyproline and glucosamine during the antiarthritic analysis of the test drug. Hydroxyproline is produced by hydroxylation of the amino acid proline by the enzyme prolyl hydroxylase following protein synthesis (as a posttranslational modification). The enzyme catalysed reaction takes place in the lumen of the endoplasmic reticulum. Although it is not directly incorporated into proteins, hydroxyproline comprises roughly 4% of all amino acids found in animal tissue, an amount greater than seven other amino acids that are translationally incorporated. Hydroxyproline is a major component of the protein collagen.

Glucosamine (C_6_H_13_NO_5_) is an amino sugar and a prominent precursor in the biochemical synthesis of glycosylated proteins and lipids. Glucosamine is part of the structure of the polysaccharides chitosan and chitin, which compose the exoskeletons of crustaceans and other arthropods, cell walls in fungi, and many higher organisms. Glucosamine is one of the most abundant naturally occurring amino monosaccharides that has been used to treat or prevent osteoarthritis in humans.

The effect of adjuvant-induced arthritis on the urinary excretion of hydroxyproline and glucosamine was investigated in rats on the 2nd, 14th, and 21st days of the treatment after the injection of CFA. The rats were kept overnight in metabolic cages on the 14th and 21st days to collect urine samples for the estimation of hydroxyproline and glucosamine. The hydroxyproline and glucosamine content in urine was also calculated in mg/mL from the absorbance at 540 nm calibration curve which was generated with different concentrations of these two compounds, respectively.

#### 2.9.4. Radiographical Examination of the Ankle Joints of the Rats

The radiographic examination of animals of all groups was done using dental X-ray plate in the probe diagnostic laboratory, Kolkata, by performing X-ray images of left and right paws on 28th day to assess swelling or other changes in the cartilages or destruction and irregular margin of the bone and cartilage in the paws.

#### 2.9.5. Histopathological Analysis of the Left Ankle Joints of Rats

This test is done to find out the degree of chronic inflammation which has occurred in the affected parts. All the animals were sacrificed at the end of the experiment on the 28th day. The left hind paws of all the animals were removed, fixed in formal saline for 7 days, and then decalcified in 5% formic acid. Joints were then trimmed and embedded and sections were cut just above the ankle joint having a thickness of 6 *μ*m by microtome. These sections were stained with haematoxylin and eosin and mounted on the slide permanently for histopathological observation to ascertain the presence of dense inflammatory infiltrate in the joints, arthritis with destruction of cartilage, pannus formation, and so forth under microscope (Dewinter, Italy).

#### 2.9.6. Histopathological Analysis of the Liver, Stomach, and Kidney

Since use of NSAIDs on long-term basis has been known to be associated with toxicity, this experiment was done to assess the pharmacological effect or any toxicity of the prescribed drugs upon the stomach, liver, and kidney of the rats in all groups after the end of the experiment. Fresh portions of lateral lobes of the liver, stomach, and kidney from each sacrificed rat were cut rapidly, fixed in neutral buffered formalin (10%), and then dehydrated with varying grades of ethanol (70, 80, 90, 95, and 100%) followed by clearing of the samples in xylene. These samples were then impregnated with 2 changes of molten paraffin wax, embedded, and blocked out. Paraffin sections (4-5 *μ*m) were stained with hematoxylin and eosin, the conventional histological stainer according to Pearse. Stained sections of control and treated rats were examined for alterations in the architecture, portal triads, hepatocytes, and sinusoids and for the presence of degeneration, necrosis, fatty change, and portal fibrosis.

### 2.10. Statistical Analysis

The data were statistically analyzed using one-way ANOVA followed by Dunnett's *t*-test for individual comparison of groups with control. Results were expressed as mean ± SD. *P* < 0.05 was used to indicate statistical significance.

## 3. Results

### 3.1. Pharmacognostical Studies

The powder of the seeds is light brown in colour with an aromatic odour and has a slightly sweet taste. The microscopic structure of the powder showed the presence of cells containing tannins, stone cells, crystals, endospermic cells, starch grains, and vessels.

### 3.2. Physiochemical and Phytochemical Analysis

The values of various important physiochemical parameters for the purpose of quality control and standardization such asmoisture content, ash value, and extractive value were evaluated following methods prescribed in the Ayurvedic pharmacopoeia as detailed in Table 1.

### 3.3. Acute Toxicity Study

The animals tested in acute toxicity tests up to the dose of 1000 mg/kg showed no significant toxic symptoms like sedation, convulsion, diarrhoea, irritation, and so forth and no signs of behavioural changes. No mortality was reported up to 24 hrs and even later during the subsequent 14 days at 1000 mg/kg dose.

### 3.4. Subacute Toxicity Study

**Figure 1:**
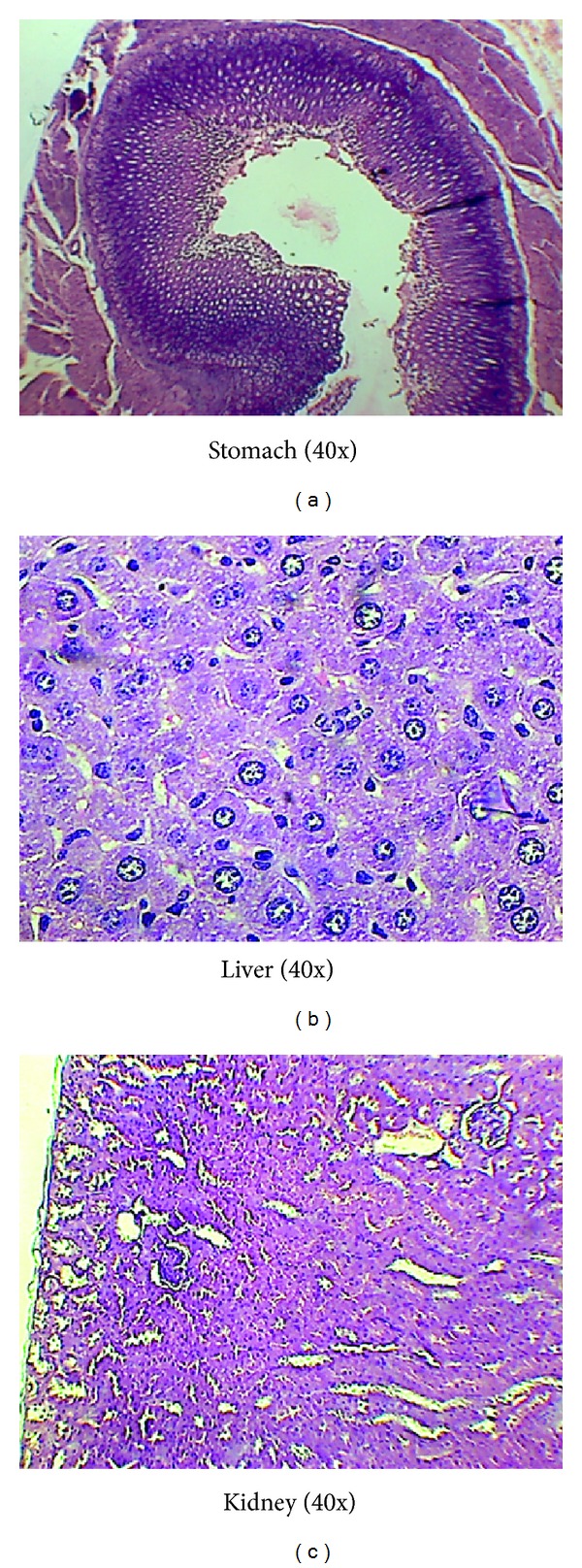
Histopathological analysis of stomach, liver, and kidney of the acetone ext up to the dose of 1000 mg/kg showing normal structures under magnification 40x during subacute toxicity.

It is observed that one rat at the dose of 900 mg and 1000 mg/kg out of 6 rats died due to some other factors during daily dose pattern toxicity. This was confirmed since no pathological changes occurred in the histopathological slides of the liver, stomach, and kidney shown below. The assessment of physical changes and behaviour activities was also recorded in the subacute toxicity test like acute toxicity analysis of this research drug (Table 2). No noticeable changes were noticed in the values obtained during the haematological test of the blood sample of treated groups up to the dose of 1000 mg/kg as compared to the normal values. The histopathological images of the stomach, liver, and kidney of the rats administered the highest dose of 1000 mg/kg showed normal internal structure of mucosal lining, hepatic cells, kupffer cells, and cell content as shown in Figures 1(a)–1(c).

### 3.5. Evaluation of Antiarthritic Activity

The values of mean body weight and left paw oedema and diameter of left paw ankle joint after daily administration of the prescribed dose were calculated over time following the adjuvant arthritis model in case of all groups. The size of right paw oedema was measured and the clinical features of inflammation were observed after 14th day and 21st day of this study because these changes may occur in this test as per protocol of the CFA reagent.

#### 3.5.1. Changes in Body Weight of the Rats over Time

**Figure 2:**
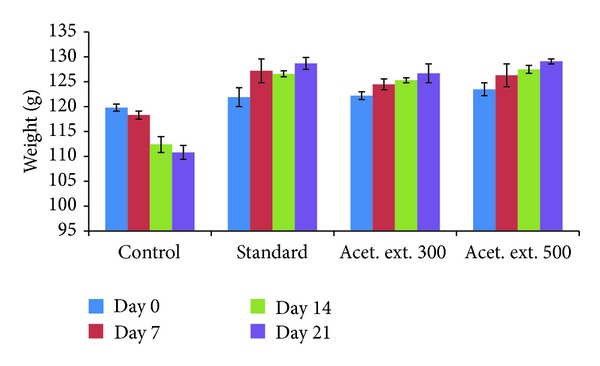
Effect of acetone extract (AE) of *Saraca asoca* on body weight.

The changes in body weight of rats were monitored up to 21 days and the results are shown in Table 3. The significant decrease in mean body weight of control group rats from 119.8 gm on day 0 to 110.8 gm on the 21st day may be due to decrease in their immune response and increased symptoms of inflammation, pain, fever, and swelling due to subplantar administration of CFA. On the other hand, the other groups showed appreciable increase in body weight during this period, ranging from 5.57% in case of the standard group to 3.68% in case of the 300 mg/kg group and 4.53% in case of the 500 mg/kg drug group (Figure 2), which may be attributed to the response of the treatment drug against the induced inflammation.

#### 3.5.2. Changes in Left Paw Diameter

**Figure 3:**
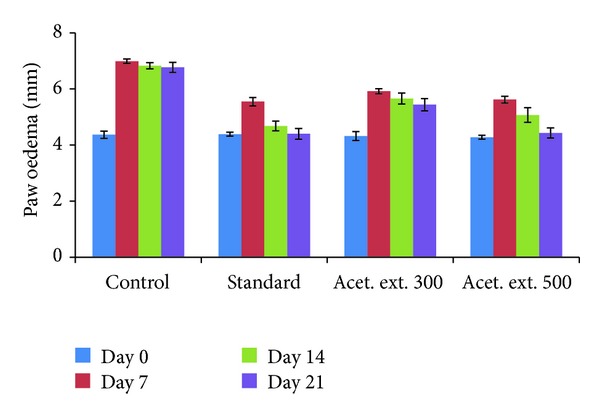
Effect of acetone extract (AE) of *Saraca asoca* on paw diameter.

The paw volume was measured in terms of the diameter in mm for all the groups and compared with the control group on the 7th, 14th, and 21st days. In case of the control group, the paw oedema increased consistently from 4.37 mm to 6.77 mm over this period. The group which was administered 500 mg/kg drug exhibited inhibition of 48.85%, 67.88%, and 93.75% as compared to the control group on the 7th, 14th, and 21st days (Table 4). These results are very close to the figures of 55.72%, 88.21%, and 99.58% obtained over the same period in case of the standard indomethacin group, signifying very strong and significant anti-inflammatory activity. Strong and sustained anti-inflammatory action was also noticed in case of the lower dose of acetone extract (300 mg/kg) where the observed inhibition values were 38.93%, 45.52%, and 53.33% after 7, 14, and 21 days (Figure 3).

#### 3.5.3. Changes in Left Foot Ankle Joint Oedema

**Figure 4:**
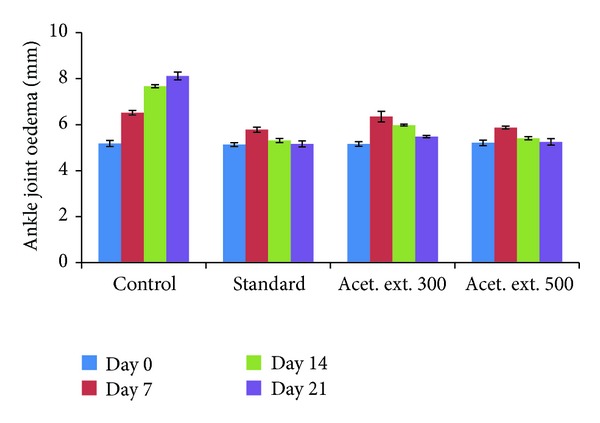
Effect of acetone extract of *Saraca asoca* on ankle joint diameter.

The left ankle joint oedema in the control group increased significantly over the 21-day period, while substantial inhibition was observed in case of the other research groups. In case of the drug extract group at higher dose, 50.75%, 91.96%, and 98.63% inhibition was noticed after 7, 14, and 21 days, respectively, as compared to the control group, indicating significantly high anti-inflammatory activity which is quite close to the corresponding values of 51.49%, 92.77%, and 98.97% in case of the standard drug group (Table 5). The drug group extract at the lower dose (300 mg/kg) also exhibited significantly high activity especially on the 14th and 21st days (Figure 4) when 67.06% and 89.11% inhibition was noticed.

#### 3.5.4. Estimation of Hydroxyproline and Glucosamine in Urine Sample of the Rats

The hydroxyproline and glucosamine content in urine was calculated in mg/mL from the calibration curve which was generated with different concentrations of these two compounds on the 2nd, 11th, and 21st days. These parameters were found to exhibit an increasing pattern in the control group due to excessive passing of connective tissues in the form of amino acid proline and amino monosaccharide in the urine which indicated that there is no anti-inflammatory effect on the induced inflammation of the body parts. However, these two metabolites showed a decreasing pattern in the other groups; the standard group exhibiting the highest decrease followed by the higher drug dose group and the lower drug dose group, respectively (Table 6).

#### 3.5.5. Effect on Haematological Parameters and Prostaglandin Level of Treated Groups

There was a significant decrease in the values of RBCs along with Hb% and significant increase in WBCs and ESR in the control group rats as compared to normal rats after 21 days of treatment which indicated that the second stage of inflammation had occurred in the body. However, the haematological parameters of the blood of rats treated with acetone extract (300 mg/kg and 500 mg/kg) and the standard drug indomethacin showed a lower decrease in the red cells counts and Hb% and also a lower increase in the WBC count and ESR values (Table 7). In comparison, it can be seen that the highest impact was seen in case of the standard group, while the lowest impact was seen in the lower dose of the acetone extract, even though significant and sustained impact was noticed in all the three groups as compared to the control group.

The anti-inflammatory effect of these three groups on the basis of the above haematological results was confirmed by the changes observed in the various groups after 21 days in the level of the prostaglandin which was found to be lower in the standard and higher drug dose treated groups as compared to the control group.

#### 3.5.6. Radiographical Scanning after Treatment with CFA

**Figure 5:**
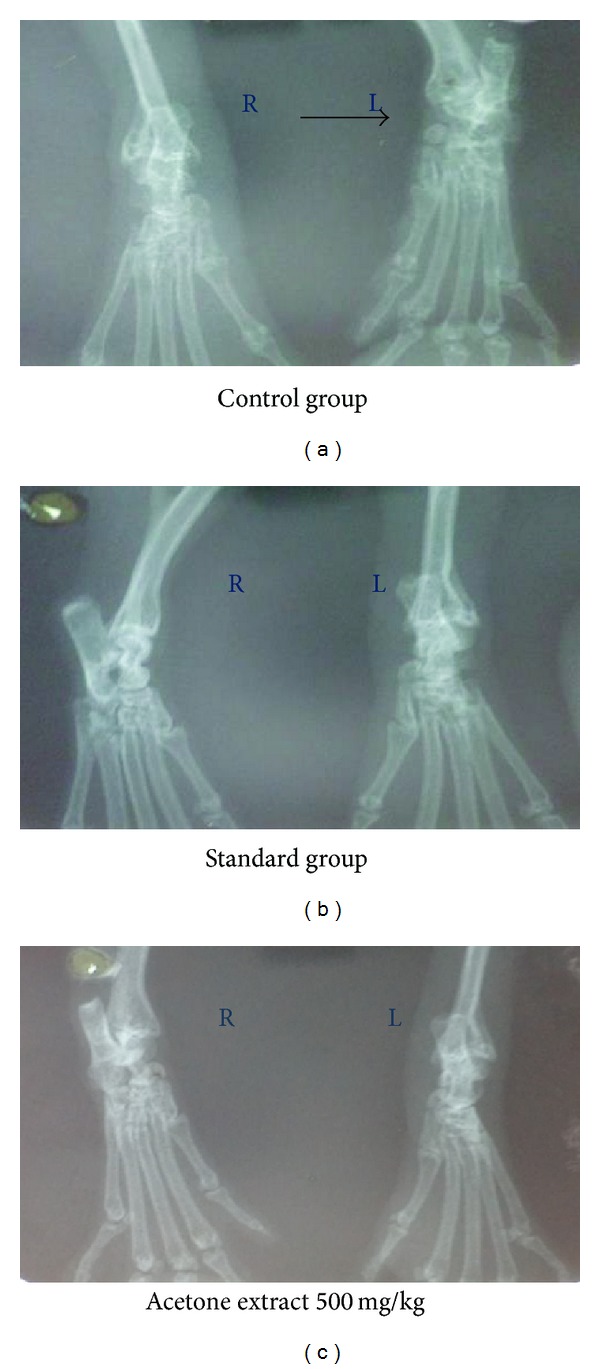
Radiographic images of left and right paw of rats after 28 days of experiment.

The X-ray image of the tibia-tarsal joints of left and right paw of adjuvant-induced control group showed marginal bone swelling and a slight destruction of the cartilage bone in the left and right paw after 28 days of treatment. The radiographic images (Figures 5(b) and 5(c)) of the adjuvant-induced standard indomethacin drug group and higher dose (500 mg/kg) of acetone extract after 28 days (Figures 5(a)–5(c)) showed no callus formation, deformity, or irregular margins of cartilage and bone, which indicated the significant pharmacological anti-inflammatory action as compared to the control group.

#### 3.5.7. Histopathological Analysis of Left Ankle Joint on the 28th Day of Experiment

**Figure 6:**
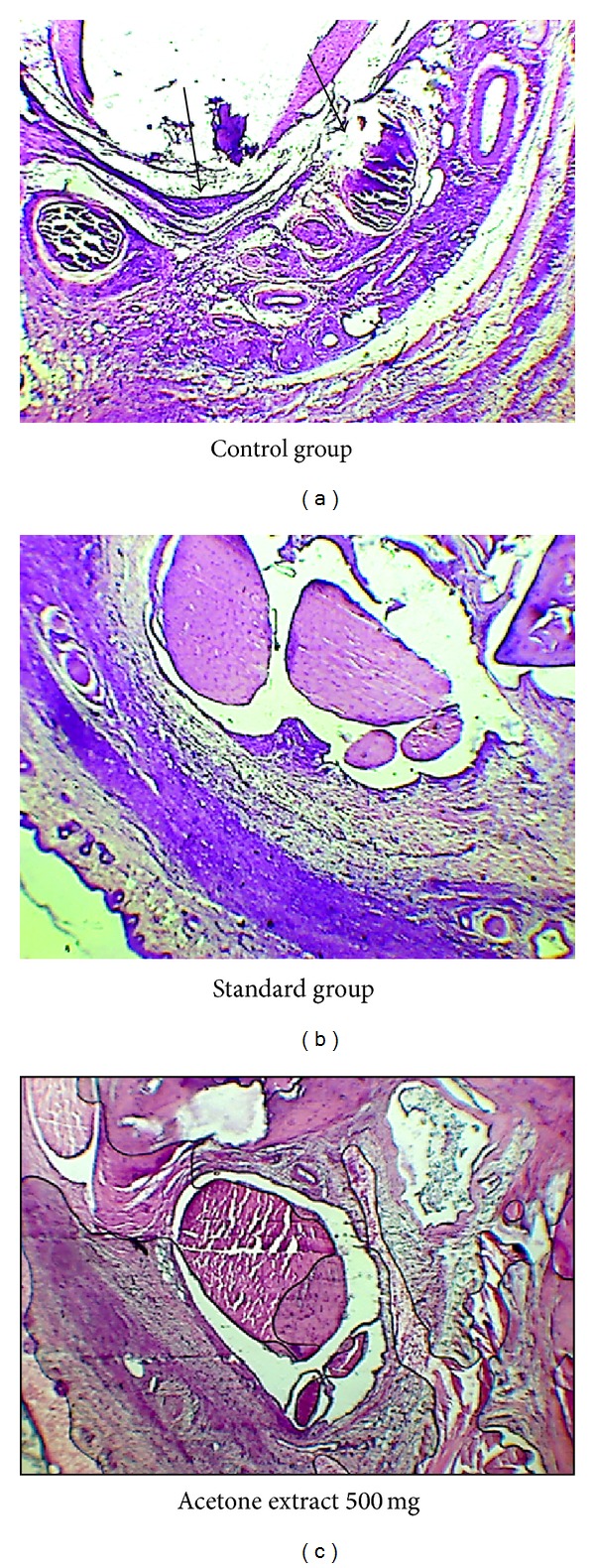
Histopathological images under Microscopic magnification 40x of the left ankle joints after 28 days showing erosion in synovial membrane and cartilage in control group.

The results of the histopathological analysis of the left ankle joints after 28 days as seen under the microscope are shown in Figures 6(a)–6(c) which reveal signs of mild bone destruction by synovial proliferation, cellular infiltration, and cartilage erosion in the control group, while no such changes are seen in the other two groups.

#### 3.5.8. Histopathological Analysis of the Liver, Stomach, and Kidney of Rats after 28 Days

**Figure 7:**
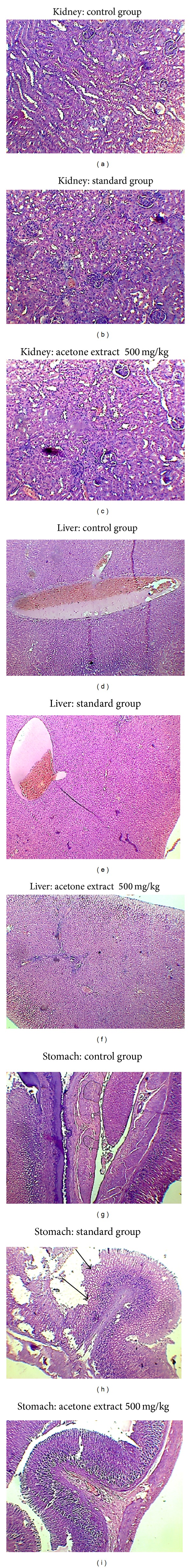
Histopathological analysis of liver, kidney, and stomach of the control, standard indomethacin, and acetone extract 500 mg/kg showing changes in the endothelium of the stomach of standard group, normal liver and kidney cell structures, and so forth in all groups under magnification 40x.

The histopathological analysis of the three important internal organs of the body, namely, the kidney, liver, and stomach, was done in order to evaluate the changes that may have occurred in their internal structure after 28 days of this study in respect of the adjuvant-induced treated rats administered with standard drug indomethacin and acetone extract at 300 mg/kg and 500 mg/kg dosage. It was observed from the obtained results shown in Figures 7(a)–7(i) that no changes or abnormal features were noticed in the slides of kidney and liver in respect of all the three groups. However, the slide of stomach of the standard drug indomethacin showed the irregular and broken margins of the endothelium of stomach, possibly indicating the initial stage of the production of ulcer because usage of NSAID drugs has been shown to be associated with such side effects in some cases. On the other hand, the slides of both doses of the acetone extract of the research drug showed the features of normal structure of the stomach of rat which indicated its safety and nontoxic effect.

## 4. Discussion

The *Saraca asoca* bark has a stimulating effect on the endometrium and ovarian tissue and is useful in menorrhagia during uterine fibroids. It also has great benefits for its uterine activity. Flowers of this tree are used to treat cervical adenitis, biliousness, syphilis, hyperdipsia, burning sensation, hemorrhagic dysentery, piles, scabies in children, and inflammation in a traditional system of medicine [21].

FCA-induced arthritis has been used as a model for assessing subchronic or chronic inflammation in rats and is of considerable relevance for the study of pathophysiology and pharmacological control of inflammatory processes [22]. During the study, the initial inflammatory response was developed within few hours, but more critical clinical signs were seen during the 1st week of postinoculation and thereafter for several weeks [19, 23].

The chemical analysis of acetone extract of the seed exhibited the presence of high concentrations of phenolic compounds such as flavonoids, tannins, carbohydrates, and acetyl salicylic acid after following the standard pharmacognostical methods. During the acute and subacute toxicity assessment of the acetone extract, there was no mortality and no other severe symptoms were observed up to the dose of 1000 mg/kg body weight of mice.

During the antiarthritis studies, the body weight of the rats after 21 days of treatment increased appreciably ranging from 5.57% in case of the standard group to 3.68% in case of the 300 mg/kg group and 4.53% in case of the 500 mg/kg drug group. At the same time, there was a decrease of 7.51% in body weight in case of the control group which may be due to the nonsubsidence of the clinical symptoms and pathological changes in the induced paw edema.

The changes in the left paw oedema volume as compared with the control as measured on the 7th, 14th, and 21st days showed that the inhibition of inflammation in case of the drug-treated group at higher dose (500 mg/kg) was comparable to that in case of the standard group and a little lower in case of the lower drug dose (300 mg/kg) group. Measurements of the ankle joint size over the same time period in case of the different groups were also compared with the control group and similar results were obtained, with the lower drug group also exhibiting very high inhibition of inflammation after 21 days. The similarity in the pattern of inhibition of the oedema in the paw and the ankle joints of the acetone extract (500 mg/kg) as compared to the standard group could be attributed to the presence of high concentrations of flavonoidic phenolic compounds in the extract which are known to exhibit antimicrobial, antiviral, antiulcerogenic, cytotoxic, antioxidant, antihepatotoxic, antipyretic, and anti-inflammatory activities.

Mild oedema in the right paw and the other clinical features were noticed in the control group after 21 days, but no similar changes were noticed in the drug and standard groups which also indicated the positive effect of drug.

The amount of hydroxyproline and glucosamine in urine as measured on the 2nd, 11th, and 21st days was found to have an increasing pattern in the control group indicating absence of any anti-inflammatory effect upon the induced inflammation. However, their concentrations showed a decreasing pattern in the other groups; the standard group exhibiting the highest decrease followed by the higher drug dose group and the lower drug dose group, respectively.

The results of the changes in the haematological parameters such as Hb%, RBC count, WBC count, and ESR levels as well as the level of prostaglandin also indicated that the maximum anti-inflammatory effect and mechanism of the action of the drug were observed in case of the standard group, but a comparable significant impact was seen in case of the 500 mg/kg drug group followed by the lower drug group. Indomethacin inhibits the catalytic activity of the COX enzymes, the enzymes responsible for catalyzing the rate-limiting step in prostaglandin synthesis via the arachidonic acid pathway.

The radiographic images obtained during the X-ray of experimental animals showed that none of the changes, namely, bony swelling, destruction of the cartilage, articular meniscus, and so forth, were exhibited in the acetone extract 500 mg/kg treated and the standard groups, while the erosion in synovial membrane and cartilage was found in the control group.

The histopathological images of the stomach, liver, and kidney showed no adverse impact upon the liver and kidney in any of the groups, while some ulcerogenic effect was noticed in the stomach in case of the standard group. The test drug showed no toxic effect upon the three main organs of the body, stomach, liver, and kidney, when administered orally for a long time (up to 21 days). This is particularly important because it is well known that NSAIDs can produce some toxic effect on these organs when used for a long time or in high doses.

Inflammation defined as a biological process characterized by redness, oedema, fever, and pain can result in locally increased production of free radicals by inflammatory enzymes, as well as the release of inflammatory mediators that promote cell proliferation and angiogenesis and inhibit apoptosis. As per the modern pathophysiology concept, fever and inflammation are produced due to exogenous pyrogens which are generally a form of microorganism such as bacteria and virus. These exogenous pyrogens act on the host cells and produce endogenous pyrogens in the form of cytokines, which are regulatory polypeptides. The endothelial cells of anterior hypothalamus release arachidonic acid metabolites when exposed to these endogenous pyrogenic cytokines. One of the arachidonic acid metabolite prostaglandins E2 (PGE2) is a very potent fever producing autacoid and also inflammation in the body. In fact, interleukin-6 is a cytokine not only involved in inflammation and infection responses but also in the regulation of metabolic, regenerative, and neural processes.

*Saraca asoka* inhibited all TFs/DNA interactions even if at different concentrations. The medicinal plant extracts exhibiting inhibitory activity on cell proliferation should undergo analysis for possible antitumor activity, while extracts displaying inhibition of TFs/DNA interactions without effects on cell growth kinetics might be employed to control TFs-dependent gene expression without cytotoxic effects, including the case of inflammatory processes involved in relevant human pathologies, such as rheumatoid arthritis and cystic fibrosis. Several transcription factors (TFs) play crucial roles in governing the expression of different genes involved in the immune response, embryo or cell lineage development, cell apoptosis, cell cycle progression, oncogenesis, repair and fibrosis processes, and inflammation. As far as inflammation, TFs playing pivotal roles are nuclear factor kappa B (NF-*κ*B), activator protein (AP-1), signal transducer and activator of transcription (STATs), cAMP response element binding protein (CREB), and GATA-1 factors. All these TFs regulate the expression of proinflammatory cytokines and are involved in the pathogenesis of a number of human disorders, particularly those with an inflammatory component [7].

The findings indicate that the acetone extract of *Saraca asoca* possesses antiarthritic pharmacological action similar to the nonsteroidal anti-inflammatory drug indomethcin due to the presence of flavonoidic compounds quercetin and gallic acid in the test drug which may be responsible for decreasing the level of cytokine and interleukin (responsible for inflammation in the body) by measuring the responsible prostaglandin in serum. Indomethacin is a nonsteroidal anti-inflammatory drug (NSAID) that reduces fever, pain, and inflammation by reducing the production of prostaglandins. Prostaglandins are autacoids that the body produces to cause fever and pain that are associated with inflammation. The decreased level of prostaglandin in the blood and other changes in the hematological parameters have also confirmed the pharmacokinetic action of the acetone extract during this study. Indomethacin blocks the enzymes that make prostaglandins (cyclooxygenases 1 and 2) and thereby reduces the levels of prostaglandins leading to reduction in fever, pain, and inflammation. A similar mechanism of action probably exists in the research drug at the dose of 500 mg/kg acetone extract due to the presence of flavonoidic compounds that are well known to possess antipyretic, analgesic, and anti-inflammatory properties.

## 5. Conclusions

The research effort makes a detailed and comprehensive assessment of the antiarthritic efficacy of the seeds of *Saraca asoca* by evaluating the impact on various diverse parameters employing standard techniques of scientific analysis associated with Freund's adjuvant-induced arthritic rat model. The findings confirmed the significant nontoxic, antiarthritic, and anti-inflammatory pharmacological effect of *Saraca asoca*'s acetone extract which is comparable to the standard drugs in a dose-dependant manner, possibly due to the presence of flavonoidic chemical compounds and the resultant lowering in the level of prostaglandin in the blood. However, further research is required for isolation and identification of the specific chemical compounds responsible for the antiarthritic effect.

## Figures and Tables

**Table 1:** Results of the physiochemical and phytochemical analyses.

Parameter	Value
Moisture content	6.50%
Ash values	
Total ash	6.70
Water soluble ash	6.00
Acid insoluble ash	0.70
pH	4.39
Fluorescence analysis	at UV 254 nm
1 M Sodium hydroxide	Dark brown
dil. Nitric acid	Greenish brown
5% Iodine	Greenish brown
5% Ferric chloride	Black
1 M Sulphuric acid	Light green
Total phenolic content	37 mg gallic acid equivalent/gm dry material
Phytochemical constituents	Carbohydrates, glycosides, tannin, flavonoids, salicylates, saponin
Extractive value	1.27%

**Table 2:** Results of the subacute toxicity study in rats.

Clinical signs	Dose in mg/kg body weight							
	300	400	500	600	700	800	900	1000
Motor activity↑	−	−	−	−	−	−	1 Died	1 Died
Motor activity↓	±	±	−	−	+	+	−	−
Colonic convulsions	−	−	−	−	−	−	−	−
Muscle spasm	−	−	−	−	−	−	−	−
Spasticity	−	−	−	−	−	−	−	−
Loss of righting reflex	±	±	+	+	+	+	−	−
Tremors	−	−	−	−	−	−	−	−
Sedation	−	−	−	−	−	−	−	−
Lacrimation	−	−	−	−	−	−	−	−
Diarrhoea	−	−	−	+	−	+	−	−
Salivation								
Viscid	−	−	−	−	−	−	−	−
Watery	−	−	−	−	−	−	−	−
Respiration								
Depression	−	−	−	−	−	−	−	−
Stimulation	±	±	−	−	+	+	−	−
Failure	−	−	−	−	−	−	−	−
Hypnosis	−	−	−	−	−	−	−	−
Anaesthesia	−	−	−	−	−	−	−	−
Drowsiness	−	−	−	+	−	−	−	−
Irritation	−	−	−	−	−	+	−	−

+ denotes present; − denotes absent.

**Table 3:** Changes in body weight of rats over time.

Treated groups	Wt (gm.) on day 0	Wt (gm.) on day 7	% Increase in Wt on day 7	Wt (gm.) on day 14	% Increase in Wt on day 14	Wt (gm.) on day 21	% Increase in Wt on day 21
Control	119.8 ± 1.57	118.3 ± 4.25	−1.25 ± 2.85	112.4 ± 1.79	−6.17 ± 2.18	110.8 ± 2.82	−7.51 ± 2.57
Standard	121.9 ± 1.85	127.2 ± 5.35**	4.34 ± 3.72	126.6 ± 2.62***	3.85 ± 1.94	128.7 ± 5.15***	5.57 ± 3.78
Acetone extract 300	122.2 ± 3.68	124.5 ± 1.40*	1.88 ± 2.14	125.3 ± 1.07***	2.53 ± 2.39	126.7 ± 1.71***	3.68 ± 2.53
Acetone Extract 500	123.5 ± 3.19	126.3 ± 2.67*	2.26 ± 1.02	127.5 ± 4.19***	3.23 ± 1.85	129.1 ± 1.16***	4.53 ± 1.94

Each value represents mean ± SD of six animals. Statistical analysis was done by one-way ANOVA followed by Dunnett's *t*-test. The values are statistically significant at three levels *P < 0.05, **P < 0.01, and ***P < 0.001 as compared to arthritic control.

**Table 4:** Changes in left paw diameter and percentage inhibition over time.

Treated groups	Paw size (mm) on day 0	Paw size (mm) on day 7	% Inhibition on day 7	Paw size (mm) on day 14	% Inhibition on day 14	Paw size (mm) on day 21	% Inhibition on day 21
Control	4.37 ± 0.29	6.99 ± 0.17		6.83 ± 0.36		6.77 ± 0.16	
Standard indomethacin	4.39 ± 0.17	5.55 ± 0.34***	55.72	4.68 ± 0.21***	88.21	4.4 ± 0.27***	99.58
Acetone extract 300	4.32 ± 0.24	5.92 ± 0.38**	38.93	5.66 ± 0.44***	45.52	5.44 ± 0.57***	53.33
Acetone extract 500	4.28 ± 0.39	5.62 ± 0.42***	48.85	5.07 ± 0.50***	67.88	4.43 ± 0.40***	93.75

Each value represents mean ± SD of six animals. Statistical analysis was done by one-way ANOVA followed by Dunnett's *t*-test. The values are statistically significant at two levels **P < 0.01 and ***P < 0.001 as compared to arthritic control.

**Table 5:** Changes in left foot joint diameter of rats over time.

Treated groups	Joint size (mm) on day 0	Joint size (mm) on day 7	% Inhibition on day 7	Joint size (mm) on day 14	% Inhibition on day 14	Joint size (mm) on day 21	% Inhibition on day 21
Control	5.18 ± 0.30	6.52 ± 0.18		7.67 ± 0.23		8.12 ± 0.28	
Standard indomethacin	5.13 ± 0.23	5.78 ± 0.26***	51.49	5.31 ± 0.51***	92.77	5.16 ± 0.13***	98.97
Acetone extract 300 (mg/kg)	5.16 ± 0.15	6.35 ± 0.21	11.19	5.98 ± 0.09***	67.06	5.48 ± 0.16***	89.11
Acetone extract 500 (mg/kg)	5.21 ± 0.37	5.87 ± 0.29***	50.75	5.41 ± 0.11***	91.96	5.25 ± 0.32***	98.63

Each value represents mean ± SD of six animals. Statistical analysis was done by one-way ANOVA followed by Dunnett's *t*-test. The values are statistically highly significant at ***P < 0.001 as compared to control rats.

**Table 6:** Hydroxyproline and Glucosamine concentration in urine.

Treated groups	Hydroxyproline content in urine (mg/mL)			Glucosamine content in urine (mg/mL)		
	2nd day	11th day	21st day	2nd day	11th day	21st day
Control	0.082 ± 0.012	0.091 ± 0.009	0.095 ± 0.008	0.079 ± 0.010	0.085 ± 0.005	0.091 ± 0.003
Standard indomethacin	0.065 ± 0.008	0.058 ± 0.004***	0.052 ± 0.002***	0.068 ± 0.005	0.052 ± 0.006***	0.044 ± 0.006***
Acetone extract 300 (mg/kg)	0.067 ± 0.008	0.062 ± 0.005***	0.058 ± 0.003***	0.088 ± 0.008	0.075 ± 0.005***	0.071 ± 0.003***
Acetone extract 500 (mg/kg)	0.076 ± 0.009	0.065 ± 0.006***	0.061 ± 0.007***	0.077 ± 0.008	0.062 ± 0.006***	0.055 ± 0.004***

Each value represents mean ± SD of six animals. ***P < 0.001 as compared to arthritic control.

**Table 7:** Effect on haematology and prostaglandin level of rats after 21 days of experiment.

Parameters	Normal	Control	Standard indomethacin	Acetone extract 300 (mg/kg)	Acetone extract 500 (mg/kg)
Hb%	14.60 ± 0.40	11.20 ± 0.21	12.20 ± 0.24	12.80 ± 0.57	12.80 ± 0.37
RBC (mil/cu·mm)	4.80 ± 0.14	4.00 ± 0.24	4.20 ± 0.20	4.05 ± 0.08	4.30 ± 0.15
WBC (/cu·mm)	6400 ± 186.33	8600 ± 161.25	6900 ± 113.67	7800 ± 169.35	7200 ± 132.97
ESR (mm)	15 ± 0.46	45 ± 0.32	25 ± 0.80	34 ± 0.63	28 ± 0.33
Prostaglandin	14.37 ± 0.32	15.05 ± 0.49	14.76 ± 0.23	15.02 ± 0.40	14.72 ± 0.32

Each value represents mean ± SD of six animals.
